# Case report and review of the literature: Successful transition from acute continuous veno-venous hemodiafiltration therapy to chronic peritoneal dialysis in a chronically ventilated child with hypoplastic left heart syndrome following fontan

**DOI:** 10.3389/fped.2022.1040869

**Published:** 2022-11-01

**Authors:** Susan D. Martin, Marc B. Lande, Joseph D. Kuebler, Jill M. Cholette

**Affiliations:** Department of Pediatrics, University of Rochester Medical Center, Rochester, NY, United States

**Keywords:** hypoplastic left heart syndrome, fontan, acute kidney injury, chronic kidney disease, renal replacement therapy, hemodialysis, peritoneal dialiysis

## Abstract

Fontan palliation depends on low pulmonary vascular resistance in order to maintain pulmonary blood flow and adequate oxygenation. This physiology results in higher central venous pressures with limited renal perfusion pressure and cardiac output. Positive pressure ventilation with mechanical ventilation increases intrathoracic pressure and raises central venous pressure and can further limit pulmonary and renal perfusion. Fluid removal with intermittent hemodialysis can be challenging in Fontan patients and can cause intolerable hypotension, however the increased abdominal filling pressures during peritoneal dialysis dwells can exacerbate systemic venous hypertension seen in Fontan patients and threaten adequate pulmonary blood flow and cardiac output. Successful transition to peritoneal dialysis in a chronically ventilated patient with hypoplastic left heart syndrome, end-stage renal disease and Fontan physiology has not been described. We present details outlining the successful transition across multiple modalities of renal replacement therapy to assist other teams faced with similar challenges in chronically ventilated Fontan patients with end-stage renal disease.

## Introduction

Total cavopulmonary connection (TCPC), or Fontan “completion” describes the cardiac surgical technique that routes systemic venous return from the hepatics and inferior vena cava (IVC) to the main pulmonary artery (PA). This third stage palliation for those with single ventricle physiology adds the IVC/hepatic blood flow to the PA and follows the second stage (bidirectional Glenn) palliation that connected superior vena cava (SVC) blood flow to the PA (1). Given the lack of a “sub”-pulmonary ventricle to draw in (*via* negative intra-thoracic pressure) or pump the systemic venous return into the pulmonary arterial vascular system, flow into the PAs is dependent on low pulmonary vascular resistance (PVR) to allow for adequate oxygenation (2). When unfavorable cardiopulmonary conditions (i.e., atrioventricular valve insufficiency, pulmonary hypertension, poor systemic ventricular dysfunction, pleural effusions) cause poor cardiopulmonary hemodynamics, TCPC can be complicated by low cardiac output, systemic and hepatic venous hypertension, protein losing enteropathy, plastic bronchitis, reduced renal perfusion pressure, renal insufficiency, fluid overload, peripheral edema, and ascites (3, 4).

Chronic positive pressure ventilation increases intrapulmonary pressures and thereby reduces the blood flow gradient of systemic venous return through the TCPC and risks adequate oxygenation (5). Peritoneal dialysis (PD) may cause increased intra-abdominal pressure from fluid instillation, further raising systemic venous pressure and central venous pressure (CVP), and therefore PA pressure with an additional limitation to pulmonary blood flow. To our knowledge there are no previous reports of successful use of chronic PD in a child with Fontan physiology and concomitant requirement of chronic mechanical ventilation. Whether PD would be hemodynamically tolerated and thereby successful in our patient with hypoplastic left heart syndrome (HLHS) status post Fontan who required chronic mechanical ventilation was unknown. Use of PD for post-Fontan patients was included in the review of acute kidney injury (AKI) and renal replacement therapy (RRT) by Niaz et al. 2021 though duration of PD was limited (median 5.5, range 3–13 days), PD details were not included, and coinciding use of mechanical ventilation not mentioned (6).

Here we present a case of successful transition from continuous veno-venous hemodiafiltration (CVVHDF) to intermittent hemodialysis (HD) to PD in a chronically ventilated child with HLHS who developed end-stage renal disease (ESRD) following Fontan palliation.

## Case presentation

**Figure 1:**
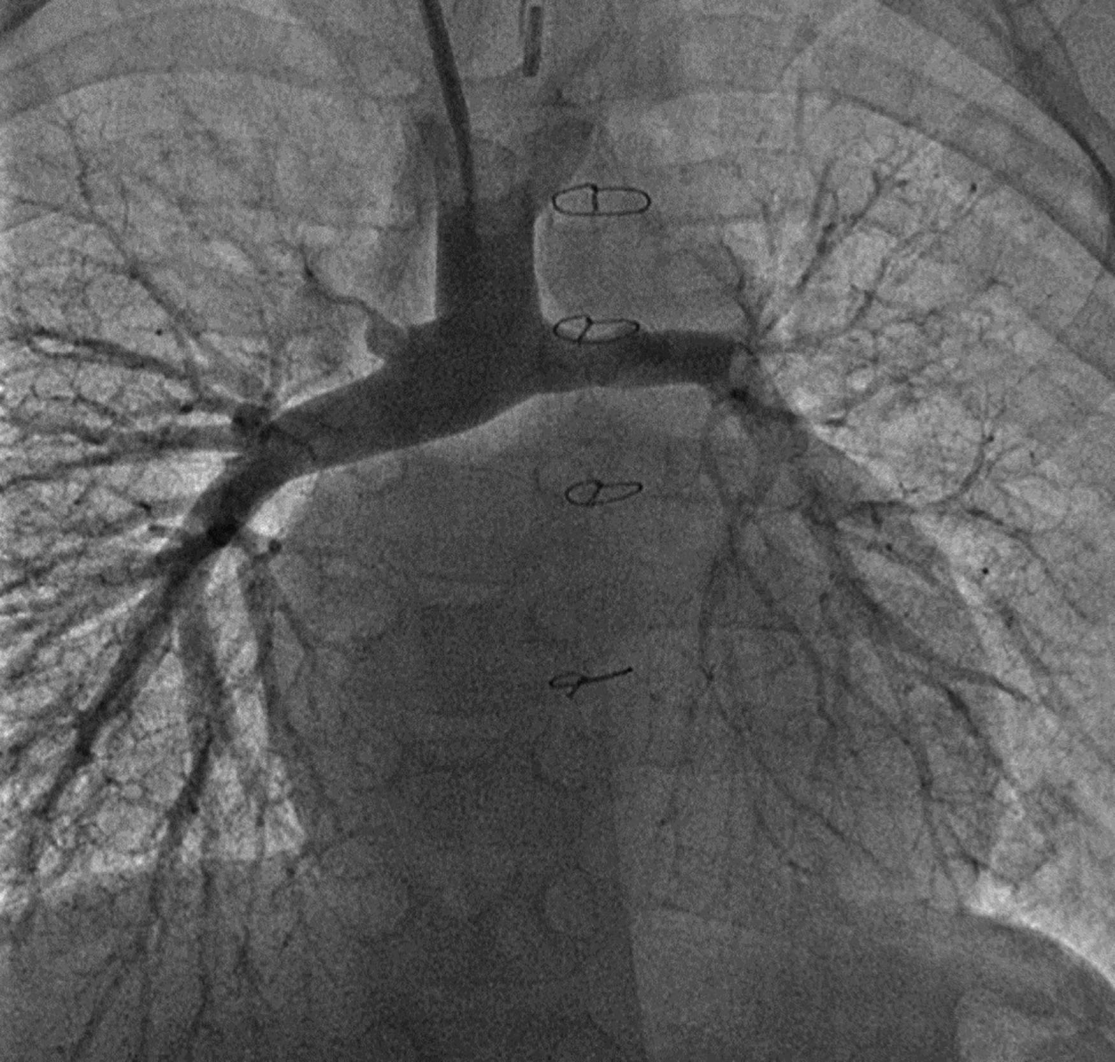


A 5-year old 19 kg boy with HLHS (mitral atresia, aortic atresia) status post Norwood/Sano and bidirectional Glenn, underwent TCPC (Fontan) palliation with a 3.8 mm fenestrated 16 mm polytetrafluoroethylene (PTFE) extracardiac conduit. His pre-Glenn course included diaphragm plication, gastrostomy tube placement and tracheostomy with chronic mechanical ventilation for subglottic stenosis and severe left bronchomalacia until he was 4 years of age. A pre-Fontan cardiac catheterization was favorable with mean bilateral PA pressures of 12–13 mmHg and no branch PA stenosis (Figure 1). His TCPC required dissection of dense adhesions but was otherwise uncomplicated. Trans-esophageal echocardiogram (TEE) post-procedure revealed a widely patent IVC anastomosis, a patent Glenn anastomosis to the right pulmonary artery, trivial tricuspid valve regurgitation (TR), and qualitatively normal right ventricle (RV) systolic function and an epicardial echocardiogram demonstrated a patent fenestration with a mean gradient of 4 mm.

**Figure 2:**
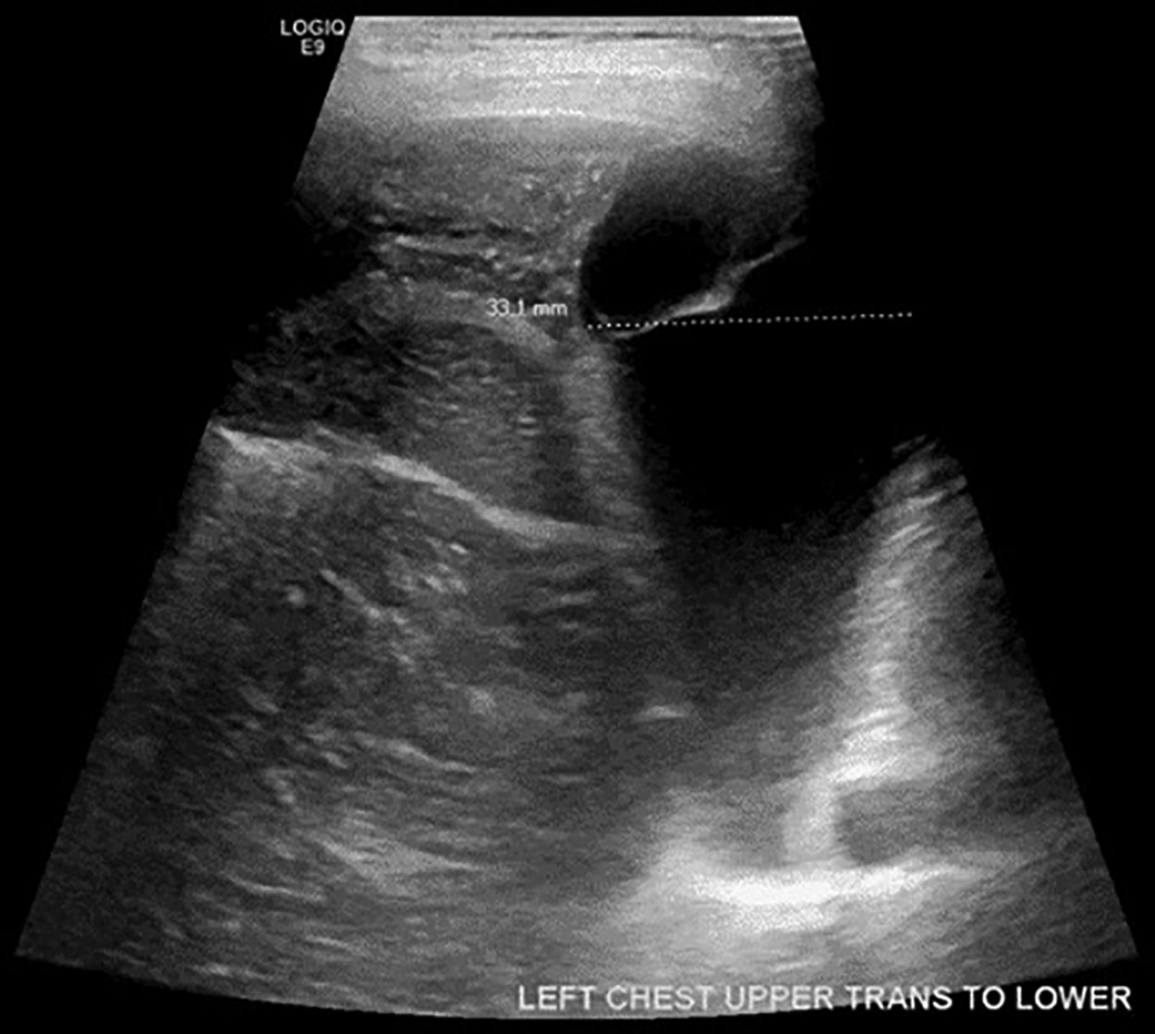


His post-TCPC course was complicated by a large chylous left pleural effusion confirmed by ultrasound (Figure 2) on POD 6. Echocardiogram (ECHO) demonstrated mild RV systolic dysfunction, mildly dilated right atrium and RV, mild TR, and mild neo-aortic insufficiency and no fenestration. Pulmonary edema and increased Fontan pressures (17–21 mm Hg) persisted despite diuretics and milrinone infusion. He subsequently developed methicillin resistant staphylococcus aureus (MRSA) sepsis and tricuspid valve endocarditis requiring intubation and escalation of vasoactive infusions, and ultimately peripheral cannulation for veno-arterial extracorporeal membrane oxygenation (VA ECMO) POD 10–11. At the time of cannulation Fontan pressures were 23–25 mmHg and he had a moderate-severely dilated RV with moderate-severely decreased RV systolic function, and moderate TR by ECHO. He was successfully decannulated after 7 days of ECMO. Fontan pressures were 21–28 mmHg following decannulation, a vegetation remained visible with persistent moderate TR. Further ECHO data is referenced in Table 1.

**Table 1:** Details outlining transition between modes of dialysis.

Event	Route of dialysis	Details/duration	CVP	Echo data
POD 20–56	CVVHDF	Continuous -over 24 h	21–26	Adequate contractility and RV function; moderate TR with improvement depending on volume status over this time period
POD 57 sildenafil added	CVVHDF	continuous -over 24 h	13–18	
POD 60	CVVHDF	20 h	19–20	
POD 61	CVVHDF	16 h		
POD 65	CVVHDF	12 h		
POD 66	CVVHDF	8 h		
POD 77–88 Hypotension; fluid removal adjusted	HD	Daily 4 h/day		Qualitatively normal RV function, mild-mod TR, no vegetation
POD 90–97 Hospital discharge POD 97	HD	4 h HD 4x/week		
One week after discharge readmission for MSSA sepsis required epinephrine drip ×4 days	HD	4 h HD 4×/week After recovery HD 3×/week		Moderately decreased RV function; mild-moderate TR; moderately dilated RV
6 mo. after hospital discharge admit for PD catheter	HD	4 h 3×/week		Mild TR, moderately decreased RV function; moderate-severely dilated RV
13 weeks after PD catheter insertion, hospitalization for transition to CCPD PD -1	CCPD	300 ml fill; last fill 0 ml 6 cycles with 54 min dwell; therapy time 6 h.		
Hospital day 2, PD -2 Hypotensive, PD paused	CCPD	300 ml fill; last fill 0 ml 12 cycles with 54 min dwell Therapy time 12 h.		
Hospital day 3; PD - 3 completed 9 cycles due to hypotension overnight on PD	CCPD	400 ml fill; last fill 0 ml 12 cycles with 54 min dwell Therapy time 12 h.		
Hospital day 5, PD - 4 Tolerated; completed; 1l fluid removal	CCPD	400 ml fill; last fill 0 ml 12 cycles with 54 min dwell Therapy time 12 h.		
Hospital day 6; PD – 5 1200 ml fluid restriction/day	CCPD	500 ml fill; last fill 0 ml 12 cycles with 4 min dwell Therapy time 12 h.		
Hospital day 7–11; PD – 6–10 HD catheter removed after 1 week of PD and 14 weeks after PD catheter insertion	CCPD	600 ml fill; last fill 0 ml 12 cycles with 54 min dwell Therapy time 12 h.		Moderate TR; moderately decreased RV systolic function; mod-severely dilated RV No pleural/pericardial effusions
Hospital day 12 Discharged home	Home CCPD	600 ml fill; last fill 0 ml 12 cycles with 54 min dwell Therapy time 12 h.		

CCPD, continuous cycler-assisted peritoneal dialysis; CVVHD, continuous veno-venous hemodiafiltration; PD, peritoneal dialysis; RV, right ventricle; TR, tricuspid regurgitation; POD, post-operative day; HD, hemodialysis; MSSA, methicillin susceptible staphylococcus aureus; CVP, Fontan pressures in mmHg.

With no response to diuretics, CVVHDF was started POD 20 for fluid overload (+11.4 L) and anuric renal failure. His prior tracheostomy site was re-cannulated for chronic mechanical ventilation. He required epinephrine (0.03–0.07 mcg/kg/min) throughout his CVVHDF course to maintain his blood pressures during that therapy. After 38 days of CVVHDF, he was hemodynamically stable off pressor support with mild tricuspid regurgitation but oliguric acute kidney injury persisted and the decision was made to transition to daily intermittent hemodialysis (HD) eventually spaced to four days a week. Given his Fontan physiology, his hemodialysis was delivered over 4 h at a relatively low blood flow rate of 120 ml/min to allow adequate fluid removal without inciting hypotension. On POD 97, he was discharged home but required readmission to the intensive care unit (ICU) for each dialysis session given his chronic mechanical ventilation and need for hemodynamic monitoring.

The patient had multiple repeated admissions for bacteremia [methicillin sensitive staphylococcus aureus (MSSA) and subsequently enterococcus], HD volume removal adjustments, and HD catheter changes. He was evaluated but determined to not be a candidate for cardiac and renal transplant. The burden of four days a week inpatient HD and the difficulties with infections led to the decision to work towards peritoneal dialysis despite the following risks: (1) concern for possible prior breach of pleural and peritoneal membranes risking hydrothorax, and (2) concern that adequate volume dwells could limit his venous return, pulmonary blood flow and respiratory function. After careful consideration between providers and his family, the decision was made to proceed and a laparoscopic PD catheter was placed (15 Fr 57 cm, single cuff, curled-tip Argyle).

After adequate time for healing of the PD catheter incision, continuous cycler-assisted peritoneal dialysis (CCPD) was initiated with slow increases in fill volumes during an inpatient ICU admission where his hemodynamics were closely monitored. Initial hypotension was tolerated and resolved without intervention. His respiratory status was stabilized with no change in his chest x-ray (CXR) or ventilatory requirement. The patient was discharged home on CCPD with fill volumes of 600 ml (about 30 ml/kg), for 12 cycles over 12 h. Details of the patient's transition between modes of dialysis are detailed in Table 1. Three years later he remains dialysis dependent and is stable on home CCPD.

## Discussion

Acute kidney injury (AKI) after complex congenital cardiac surgery with cardiopulmonary bypass is common and associated with increased morbidity and mortality (7, 8). AKI following Fontan is associated with reduced urine output, increased colloid administration, increased chest tube duration, and hospital length of stay. Patients with prolonged mechanical ventilation are also more likely to develop AKI (9). The incidence of AKI following TCPC or Fontan completion surgeries is even more common (42%–52%) with reduced renal perfusion pressure and higher central venous pressures as significant risk factors (9, 10). Lower renal perfusion pressure is associated with more severe post-operative AKI in Fontan patients (11, 12). The etiology of decreased renal perfusion pressure in postoperative Fontan patients has not been clearly defined, however it is thought that the increase in CVP causes increase renal venous pressure (renal vein hypertension/congestion), in combination with either or both low cardiac output and systemic hypotension that results in decreased renal arterial pressure, therefore decreasing renal perfusion pressure (9, 11, 13). Systemic blood pressure may play a more important role in the development of AKI that CVP, it is postulated that patients either have a critical mean arterial pressure threshold and/or loss vs. inappropriate autoregulation that increases their risk of developing AKI (9, 10). Elevated systemic venous pressure (or CVP) in Fontan patients is multifactorial with contributing factors including: venous stasis from lack of pulsatile blood flow, reduced venous capacitance, elevated PVR, systemic ventricular systolic and diastolic dysfunction, atrioventricular valve regurgitation, anatomic obstruction and dysrhythmias (2, 14).

The incidence of RRT in patients with Fontan physiology was 6.2% in a review of 1,166 patients from 1,973 to 2017 (6). Requirement of RRT was associated with increased mortality and ICU length of stay. Nearly half of Fontan patients needing RRT underwent Fontan takedown or revision. Details regarding renal function after re-operation were not provided. Of the study population, 10% had a fenestration, with no difference in AKI development compared to unfenestrated subjects. Our patient's candidacy for catheter intervention, Fontan takedown or revision was discussed in a multidisciplinary congenital cardiac surgical conference. His prolonged bacteremia with ongoing polymicrobial positive cultures and marked inflammatory state, and earlier blood product utilization were included in risk assessment with his parents electing not to proceed with invasive interventions.

The impact of a patent Fontan fenestration on renal perfusion and AKI has not been described (15–17). A 2019 meta-analysis did not identify that an open fenestration mitigated the risk of Fontan failure, length of hospitalization, or mortality (10). At least half of young adults that undergo Fontan conversion (from initial atrio-pulmonary to cavo-pulmonary connection) develop AKI (18), however it is unknown whether or not conversions were of any benefit to young patients with AKI at the time of their initial Fontan palliation. Additional studies exploring the benefits of fenestration on AKI and description of renal function and/or recovery following Fontan take down is needed.

RRT for either acute or chronic kidney disease is particularly challenging in the Fontan patient with significant physiologic obstacles to both intermittent HD and PD techniques. Intermittent HD is often not tolerated due to HD's requirement for adequate preload and its creation of large intravascular fluid shifts that can result in significant hemodynamic instability. Case reports of HD in Fontan patients demonstrating the success of HD has been limited to those with normal ventricular function and absence of significant extracardiac disease (19). PD raises intra-abdominal pressure increases inferior vena cava and intrathoracic pressure resulting in CVP elevation (20). The very process of filling and holding fluid in the peritoneal cavity, by definition, raises intra-abdominal pressure (21). The increase in intra-abdominal pressure raising CVP decreases venous return (22) risking inadequate pulmonary blood flow potentially resulting in refractory hypoxia and impaired cardiac output in Fontan patients without fenestrations.

Intra-operative placement of peritoneal dialysis catheters at the time of cardiac surgery has been adopted at some surgical centers to prevent and manage fluid overload that commonly occurs in the early post-operative period (23, 24). Passive peritoneal drainage from PD catheters placed intra-operatively during Fontan palliations has been described retrospectively, however was associated with longer duration of mechanical ventilation even despite intra-abdominal filling with dialysate not being employed (25).

A 2021 retrospective review of 465 children undergoing TCPC in a more modern era (2010–2019) found AKI [per pediatric Risk, Injury, Failure, Loss, and End-stage renal disease (pRIFLE) criteria] in 164 (35.3%) that persisted until POD 7–9 in 72 (15.5%). Those with persistent AKI had lower renal perfusion pressure, longer duration of mechanical ventilation and higher rates of RRT. Forty-nine (10.5%) received RRT, initiated when urine output was <1 ml/kg/h and there was no response to furosemide infusion for 48 h (12). Of the 49 treated with RRT, however, only 8 were in the AKI injury/failure group with 14 in the at-risk for AKI, and 27 in the no-AKI group. It appears that RRT was utilized therefore mainly for fluid overload refractory to diuretics. RRT in this study included both peritoneal dialysis and blood filtration with additional details not provided. Timing of PD catheter placement was also not described. Unfortunately, these are all reports of management of AKI, where as we report transition to end-stage renal disease care and chronic RRT need.

A study of 81 adults with Fontan physiology found a 46% incidence of chronic kidney disease when defined using estimated glomerular filtration rate and albuminuria (26). An observational cohort study of 3,600 children with congenital heart disease found 1% of children undergoing cardiac surgery progress to end stage renal disease (ESRD) (27). Children who received dialysis for AKI during their index cardiac surgery are at a five-fold increased risk for ESRD. Those with HLHS patients at greatest risk for ESRD (>200 per 10,000 person-years) and the highest mortality. Reviews of long-term renal function after Fontan palliation outline knowledge gaps regarding the prevalence of severe chronic kidney disease and incidence of end stage renal disease (28). Fontan patients with inadequate cardiac function causing renal and/or hepatic failure are traditionally evaluated for cardiac transplant. There is a paucity of literature outlining chronic RRT for those that are not candidates for transplant let alone any report of Fontan patients managed with chronic peritoneal dialysis, nor with concurrent mechanical ventilation.

Although our patient tolerated HD, its requirement to be performed as an inpatient led to the decision to attempt PD. In our patient's case, repeated sternotomies, multiple mediastinal tubes, extensive dissection of adhesions and gastrostomy tube placement were thought likely to have resulted in peritoneal membrane adhesions and fibrosis, potentially compromising the integrity of his peritoneum. This raised concern whether his peritoneum, in the setting of increased intra-abdominal pressure, would sufficiently prevent small solute and free water transport into his pleural spaces as hydrothorax is a rare, but well described complication of PD (29). Whether increased intra-abdominal pressure would further increase his intrathoracic pressure and limit his pulmonary blood flow and cardiac output, all further compounded by his requirement for positive pressure ventilation, was unknown. Previous studies have attempted to retrospectively determine the impact of prior abdominal surgery on peritoneal membrane function and PD technique survival, however limited statistical power prevented solid conclusions and studied patients were those already receiving PD (30). Furthermore, those with previous repeated cardiothoracic surgeries in addition to abdominal surgeries were not included. It is likely that this clinical scenario is common in children and young adults with complex congenital cardiac disease following multiple cardiac and abdominal surgeries, and understanding this complex issue is important when communicating between multiple care teams and with patients and families when making decisions regarding PD.

Despite his complex medical challenges, our patient was able to tolerate daily increases in his PD filling volumes with the associated increase in intra-abdominal pressure, central venous pressure and intrathoracic pressure and maintain adequate pulmonary blood flow and therefore cardiac output. Careful coordination of his care by his cardiac intensive care, cardiology, nephrology and surgery provider teams allowed for successful transition across renal replacement strategies that eventually allowed for delivery of care at home. We hope this management outline may assist other care teams when faced with similar clinical situations in these uniquely vulnerable patients.

## Data Availability

The original contributions presented in the study are included in the article/Supplementary Material, further inquiries can be directed to the corresponding author/s.
